# Neutralization of HMGB1 improves fracture healing and γδ T lymphocyte counts at the fracture site in a polytrauma rat model

**DOI:** 10.1186/s40634-022-00453-3

**Published:** 2022-02-28

**Authors:** Preeti J. Muire, Joshua J. Avila, Alicia L. Lofgren, Joseph C. Wenke

**Affiliations:** Combat Wound Care Department, US Army Institute of Surgical Research, JBSA Fort Sam, Houston, TX 78234 USA

**Keywords:** Delayed fracture healing, Severe trauma, Osteoimmunology, DAMPs

## Abstract

**Purpose:**

Delayed fracture healing is a common consequence of polytrauma (PT) occurring in patients with multiple injuries. We believe that when early release of high mobility group box 1 (HMGB1) molecules from necrotic tissues exceed their normal levels in blood, they dysregulate immune responses associated with normal healing. This study investigates the detrimental effect of such dysregulate immune responses by targeting HMGB1 in a PT rat model with debilitating injuries. We hypothesized that neutralization of extracellular HMGB1 immediately post-trauma would ameliorate local immune dysregulation and improve fracture healing in a PT rat model.

**Methods:**

PT rats received a single dose of either anti-rat HMGB1 polyclonal antibody (PT-Ab HMGB1) or IgY isotype (PT-IgY), were left untreated (PT-C), or had a single injury/osteotomy only (OST). Fracture healing was evaluated by micro-computed tomography (µCT) and histology at 5 weeks; and macrophages and T cell counts within the fracture site were determined with flow cytometry  at 1 week.

**Results:**

Notably, bone regeneration within the fracture site in PT-Ab HMGB1 rats was improved with comparable connective tissue organization than PT-C rats. Further, only γδTCR^+^ T cells, but not macrophages and CD4^+^ and CD8^+^ T cells, were diminished at the fracture site in PT-C and PT-IgY rats. Interestingly, the PT-Ab HMGB1 rats had increased γδTCR^+^ T cells compared to PT-C and PT-IgY, suggesting their potential role in regulating fracture healing.

**Conclusions:**

Therefore, the initial burst of systemic HMGB1 following trauma may have a role in regulating bone regeneration via the modulation of a subclass of T cells within the fracture site, suggesting it’s importance as a therapeutic target in PT to combat immune dysregulation and delayed fracture healing.

**Supplementary Information:**

The online version contains supplementary material available at 10.1186/s40634-022-00453-3.

## Introduction

Fracture healing is an intricate process, and a well-balanced immune response is integral to bone repair and remodeling of complicated and straightforward fractures [1, 2]. Polytrauma (PT) injuries sustained on the battlefield are associated with extensive tissue necrosis, elevated damage associated molecular patterns (DAMPs), immune dysregulation, and delayed fracture healing or nonunion depending on the injury severity [3]. Presently, with the current standard of care there does not appear to be a discernable improvement in fracture healing in combat casualties and it remains the highest priority in military research.

High mobility group box protein 1 (HMGB1) is a potent DAMP that mounts early systemic and local inflammation following polytraumatic injuries [4–6]. When present at elevated levels, HMGB1 activates pathways that potentiate further tissue necrosis that further increases HMGB1 levels in a positive feedback mechanism, thereby causing detrimental outcomes such as cellular exhaustion and impaired healing [6, 7]. Increased extracellular HMGB1 expression has been reported in several sterile injury models, including collagen-induced arthritis or during the spontaneous development of arthritis in mice [8]. Systemic administration of neutralizing HMGB1 antibody significantly ameliorated these autoimmune diseases, indicated by reduced weight loss and diminished cartilage and bone destruction in arthritic joints [8, 9]. Another study highlighted that HMGB1 induced osteoclastogenic bone destruction associated with oral squamous cancer, whereas blocking of the HMGB1:RAGE/TLR4 axis inhibited bone destruction in a mouse model [10]. Despite the well-documented detrimental role of HMGB1 as a mediator of normal remodeling and inflammatory bone loss in simple fractures [11, 12], the role of HMGB1 in the context of PT fractures has not been elucidated. We suppose that while HMGB1 may have a role in regulating fracture repair, it may function in a dose-dependent manner. Thereby we postulate that targeting the initial burst of HMGB1 during the early minutes to hours post-injuries may be a promising approach to regulate its systemic levels throughout fracture healing.

HMGB1 induces lymphocyte activation and the cytokines secreted by lymphocytes harboring within the fracture microenvironment affect bone metabolism [13, 14]. Previous studies that elucidated the role of the adaptive immune system in fracture healing found that the lack of lymphocytes led to accelerated bone mineralization with overall deteriorated bone quality in RAG1^−/−^ knockout mice, suggesting a regulatory role of lymphocytes in fracture repair [15–17]. Further, the osteogenic role of T cells was elucidated in the context of extracellular matrix and collagen deposition in fractures [18]. Among the CD3^+^ lymphocytes, the CD4^+^ T cells are thought to have an osteogenic role via the activation of mesenchymal stem cells (MSCs) [19]. Moreover, emerging data indicate that innate lymphocytes, particularly the γδT cells, may also be necessary for bone regeneration [20]. γδT cells are early responders and can become activated during the acute phase post-trauma in response to DAMPS, thereby facilitating inflammation, chemotaxis, and cell lysis via the production of cytokine and growth factors [21]. This study investigates the detrimental effect of dysregulated immune responses mounted by HMGB1 in a PT rat model, with a focus on fracture repair and early cellular dynamics of macrophages and T cells at the fracture site. We hypothesized that neutralization of extracellular HMGB1 with a single dose of anti-HMGB1 antibody immediately post-trauma would ameliorate immune dysregulation and improve fracture repair in a PT rat model.

## Materials and methods

### Animals and surgical care

Male Sprague–Dawley (SD) rats aged 14.2 ± 1.2 weeks with the average weight of 372.5 g ± 19.2 g were used in this study. All rats were housed in a specific pathogen-free facility, provided with unlimited access to food and water, and unrestricted activity before and after all procedures. Research was conducted in compliance with the Animal Welfare Act, the implementing Animal Welfare regulations, and the principles of the Guide for the Care and Use of Laboratory Animals, National Research Council. The Institutional Animal Care & Use Committee (IACUC) at the United States Army Institute of Surgical Research approved all research conducted in this study. IACUC approval number is A-16–044 and date is June 28^th^, 2016. The facility where this research was conducted is fully accredited by the AAALAC. A pre-surgical dose of Buprenorphine SR-LAB (1.2 mg/kg, subcutaneously) was administered for pain management at least 15 min before surgery. At surgery, rats were anesthetized and maintained with 1–3% isoflurane and oxygen delivered via a nose cone on a Bain circuit connected to the rodent gas anesthesia machine (VetEquip Inc., Pleasanton, Ca). Naïve rats were not surgically manipulated and served as baseline controls. Post-surgery, rats did not receive any prophylactic antibiotics but were assessed closely for signs of distress and body weight. Rats with ≥ 10% of body weight loss relative to pre-surgery weight received 3 ml sterile surgical saline once daily subcutaneously. At the designated endpoints, i.e., 1 or 5 weeks, rats were anesthetized and humanely euthanized by cardiac exsanguination or an intracardiac (IC) overdose of sodium pentobarbital. Following euthanasia, the tissues were harvested for downstream processing. This study was carried out in compliance with the ARRIVE guidelines [22].

### Surgery

Rats were divided into two cohorts of trauma representing single 3 mm femoral osteotomy (OST)/normal and polytrauma (PT)/delayed fracture repair models. PT rats underwent three traumas, i.e., a 3 mm femoral osteotomy, a blunt chest contusion with a 0.3 kg weight dropped from a height of 68 cm to exert ~ 2 J of energy on the rat's chest, and a 100 °C water scald burn for 10 s of approximately 20% of the total body surface area. All trauma and surgical procedures were previously described in detail and can be found in [23].

### Anti-HMGB1 antibody and IgY isotype antibody injections

Immediately post trauma procedures, sub-cohorts of PT rats designated as PT-Ab HMGB1, PT-Ab HMGB1 3 × and PT-IgY, respectively, received either a single dose (1 dose/day 0) or three consecutive doses (1dose/day 0, 1, and 2) of chicken anti-HMGB1 neutralizing polyclonal antibody or isotype control chicken IgY antibody (Shino-test, Tokyo, Japan; 2 mg/kg per dose, intraperitoneal (IP)). PT rats which did not receive antibody injection were designated as PT-C. All rats were recovered in clean cages with continued monitoring.

### Blood collection and processing for ELISA

Blood was collected at 1 week post-trauma (wpt) from anesthetized rats (*n* = 4–5/OST, PT-C, PT-IgY and PT-Ab HMGB-1) via tail vein cannulation in pre-weighed EDTA tubes and centrifuged at 1000xg for 10 min to separate the plasma for HMGB1 protein quantification. Plasma was stored at -80 °C until used for the HMGB1 ELISA kit (IBL International), per manufacturer’s instructions.

### Micro-computed tomography (μCT)

At 5 wpt, OST, PT-C, and PT-Ab HMGB1 rats (*n* = 10–11) the right femur was harvested without disturbing the callus, an ex vivo radiograph was acquired (Faxitron UltraFocus), and was then fixed in 10% neutral buffered formalin followed by µCT scans (VivaCT40, SCANCO Medical). The osteotomy site was scanned at high resolution with a 10.5 μm voxel size at a voltage of 70 kVp, a current of 114 μA, and an integration time of 300 ms. Images were converted to 8-bit bitmap files using Image J (National Institutes of Health, Bethesda, MA) and reoriented with DataViewer (Bruker-MicroCT, Kontich, Belgium) so that the slices were in the plane of the defect border. Regions of interest (ROI) and three-dimensional analysis were completed using CTan (Bruker-MicroCT, Kontich, Belgium). The volume of interest (VOI) extended ~ 2.6 mm (251 slices) and was centered within the osteotomy. For the values entitled 'Defect,' a polygonal ROI was drawn around the portion in which an intact cortex was first seen, proximal and distal to the defect, and interpolated throughout the slices to simulate the area in which the intact femur should be present. For the values entitled 'Continuous Callus,' an ROI was drawn tightly around any new callus formation that extended from the cortex (any islands of bone not connected to the cortex were excluded). A new ROI was drawn every 25 slices, or more as needed, based on the rapidity of change in callus shape, and interpolated. A global threshold was calculated using the Otsu method [24], and a value of 107 was determined to be the delineation between non-mineralized and mineralized tissue. The three-dimensional image reconstructions were used to quantify bone. Values are reported as either bone volume fraction, which is the ratio between bone volume and total volume within the given VOI (BV/TV, %), or volume of bone (mm^3^). These measurements were calculated in the three cohorts, as previously described [23].

### Histology of femoral defect

Femurs (*n* = 4–8/OST, PT-C, and PT-Ab HMGB1) were harvested and processed for histological analysis. Briefly, femurs were fixed in 10% neutral buffered formalin, rinsed in type 1 ultra-pure water, decalcified in formic acid for 21 days and embedded in paraffin. Longitudinal sections of 8 μm thickness were stained with hematoxylin and eosin (H&E) or Masson trichrome (MT), and estimation of bone formation was obtained by bright field microscopy with an Olympus BX41 microscope and images were captured using CellSens standard software (Olympus). The scoring key used to assess callus quality is as follows: 0—Bony union; 1 – Callus is composed primarily of new bone (> 50%); 2—Callus is composed of fibrous tissue (> 50%), new bone (< 50%), skeletal muscle and/or adipose tissue; 3—Callus is composed of fibrous tissue (< 50%), new bone (< 50%), skeletal muscle and/or adipose tissue; 4—Callus is composed of acute hemorrhage, spindle cells and inflammatory cells (new bone present); and 5—Callus is composed of acute hemorrhage, spindle cells and inflammatory cells (no new bone). The scoring key used to assess the extent of hemosiderin-laden macrophages present at the fracture site is as follows: 0 – Absent; 1—Acute hemorrhage only; 2—Chronic hemorrhage only (with hemosiderin-laden macrophages); and 3—Acute and chronic hemorrhage. The scoring key used to assess the presence of osteoblasts and osteoclasts in the bone samples is as follows: 0 – None; 1 – Minimal; 2 – Mild; 3 – Moderate; 4 – Marked; and 5 – Severe, similar to previously published [23]. All slides were reviewed and scored by a board-certified veterinary pathologist evaluating the callus quality, the presence of hemosiderin-laden macrophages, osteoblasts and osteoclasts, cellular composition and collagen organization.

### Flow cytometry

At 1 wpt, the fracture hematoma or soft callus was collected from the osteotomised femur (*n* = 4–5/OST, PT-C, PT-IgY, and PT-Ab HMGB1) accompanied by a rinse of the area surrounding the defect with 10 ml of sterile PBS. A single-cell suspension was prepared for flow cytometry. The fracture site's cell collection process was standardized across all the animal groups in this study except for the naïve rats whose whole bone marrow cells were collected from the uninjured femur. Fracture site specimens were passed through a 40 µm filter to prepare a single-cell suspension. Cells were washed in FACS buffer and RBCs were lysed. Cells were resuspended in FACS buffer and counted by trypan blue exclusion method using the automated cell counter (Countess, Invitrogen), and 1 × 10^6^ cells/sample were stained. All wash steps were performed at 500xg for 3 min at 4 °C. Cells were stained with a live/dead stain, zombie violet dye (BioLegend), for 10 min at 4 °C and washed with PBS. The cells' Fc regions were then blocked with an anti-rat CD32/Fc block antibody before staining with the respective antibodies (Table 1). Cells were fixed with fixation buffer (R&D systems) and washed twice with FACS buffer before proceeding with permeabilization step and intracellular staining. Data was acquired on a MACS Quant Analyzer 10 (Miltenyi Biotech). All antibodies were titrated before application. Appropriate isotype control antibodies, fluorescence minus one (FMOs), and single stained cells were used as controls for appropriate gating strategies. Compensation was performed with either single stained cells or beads to ensure there was no spillover within channels. Data were analyzed using Flow logic software (Miltenyi Biotech).

**Table 1:** Antibodies and their titrated dilutions used for flow cytometry

Antibodies	Vendors	Dilution per sample
Anti-rat CD32/Fc block antibody	BD Bioscience	1:50
Anti-rat CD3 antibody	Miltenyi Biotech	1:50
Anti-rat CD4 antibody	Miltenyi Biotech	1:10
Anti-rat CD8a antibody	Miltenyi Biotech	1:10
Anti-rat γδTCR antibody	BioLegend	1:50
Anti-rat CD45 antibody	BD Biosciences	1:80
Anti-rat CD68 antibody	Miltenyi Biotech	1:10
Anti-rat CD86 antibody	Miltenyi Biotech	1:10
Anti-CD163 antibody	Novus Biologicals	1:150
Zombie violet	BioLegend	1:2000

### Statistical analysis

Statistical analyses were performed using GraphPad Prism version 8.0.0 for Windows, GraphPad Software, San Diego, California USA. Data were assessed for normality using the Q-Q plot, homoscedasticity plot, residual plot, D’Agostino and Pearson test, Shapiro–Wilk test, and the difference between means and median. ELISA data was assessed by Kruskal–Wallis test followed by Dunn’s multiple comparison test. Bone regeneration within segmental defect of OST, PT-C and PT-Ab HMGB1 cohorts was assessed by ordinary one-way ANOVA with Dunnett’s multiple comparison test for normally distributed data or Kruskal–Wallis test with Dunn’s multiple comparison test if the data was not normally distributed. Flow cytometry  data from blood was analyzed for statistical significance by performing a one-way ANOVA and Tukey’s Post-hoc analysis at *a* < 0.05 level of significance. Statistical significance was set at *p* < 0.05.

## Results

### ELISA to validate neutralization of extracellular HMGB-1 in circulation

**Fig. 1:**
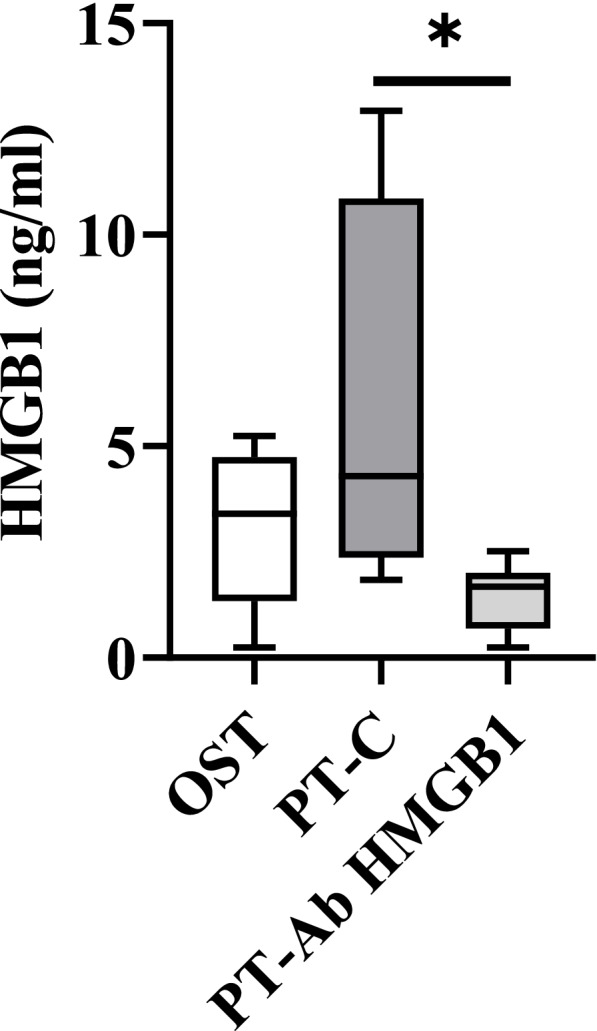
Quantitative expression levels of HMGB1 proteins at 1 week post-trauma (wpt) in circulation by ELISA. HMGB1 level were evaluated in Osteotomy (OST), Polytrauma (PT-C), and Polytrauma + Ab HMGB1 (PT-Ab HMGB1) rats (*n* = 4–5/group). Whiskers represent minimum and maximum values; and line represents median

HMGB1 protein levels were quantified by ELISA in plasma from OST, PT-C, and PT-Ab HMGB1 rats at 1 wpt. Increased HMGB1 levels were observed at 1 wpt in PT-C vs. OST and, statistical significance was noted in PT-C vs. PT-Ab HMGB1 (*p* = 0.03) (Fig. 1).

### Micro-computed tomography (μCT) to evaluate bone regeneration

**Fig. 2:**
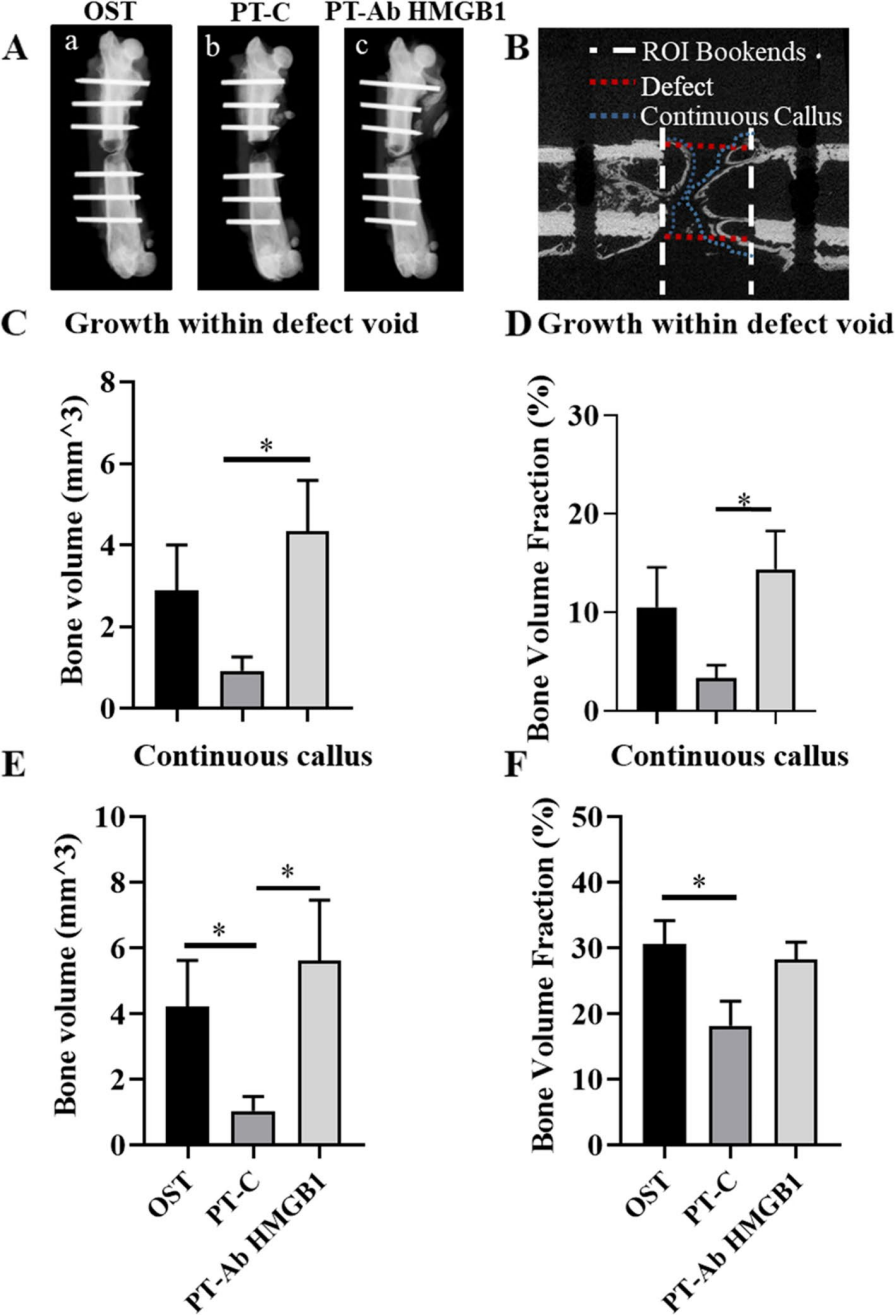
Micro-computed tomography (μCT) analysis indicates improved bone regeneration in PT following neutralization of extracellular HMGB1. (A) Representative radiographs at 5 weeks post-trauma (wpt); a) Osteotomy, b) Polytrauma, c) Polytrauma + AbHMGB1 (*n* = 10–11/group). (B) Representation of the two VOIs analyzed depicting the space in which the excised bone used to be i.e., ‘Defect’ and the volume in which any new callus has formed i.e., ‘Continuous Callus’. (C) Bone Volume (mm^3) results from the Defect VOI; (D) Bone Volume Fraction (%) results from the Defect VOI; (E) Bone Volume (mm^3) results from the Continuous Callus VOI; (F) Bone Volume Fraction (%) results from the Continuous Callus VOI. * *p* < 0.05 versus corresponding non-treated PT-C group. Data are graphically represented as mean ± SEM

To determine the role of HMGB1 in the regenerative process after PT, we assessed bone regeneration by evaluating bone healing with radiographic imaging as shown in Fig. 2A, and next measuring the amount of new bone formed in the fracture site with μCT at 5 wpt (Fig. 2B-F). Using the defect and continuous callus VOIs as described in the methods section, both the amount of mineralized tissue present (BV) and the ratio of mineralized tissue to the total volume of the VOI (BV/TV) were compared across the three cohorts, i.e., OST, PT-C, and PT-Ab HMGB1/ PT-Ab HMGB1 3x. In a pilot study, PT rats received either a single dose (PT-Ab HMGB1) or three doses (1dose/day) (PT-Ab HMGB1 3x) of anti-HMGB1 antibody. Results suggest that PT-Ab HMGB1 rats had a mean threefold increase in bone growth compared to PT-C (Fig. 2D). Whereas, the PT-HMGB1 3 × rats had a mean ~ onefold increase in bone growth (Supplementary Figure S1). For this reason, we proceeded with the single dose treatment for the entire study. Within the defect VOI, the bone volume and bone volume fraction in PT-Ab HMGB1 rats increased significantly compared to PT-C rats (*p* = 0.006 and *p *= 0.01, respectively). Further, within the continuous callus VOI, the PT-C average bone volume was significantly decreased compared with both OST (*p* = 0.02) and PT-Ab HMGB1 (*p *= 0.009). In comparison, the bone volume fraction was only different between OST and PT-C (*p* = 0.02) but not PT-Ab HMGB1 (*p* = 0.06). In both VOIs, there were no significant differences between PT-Ab HMGB1 and OST (Fig. 2C-F).

### Histological evaluation to confirm bone regeneration

**Fig. 3:**
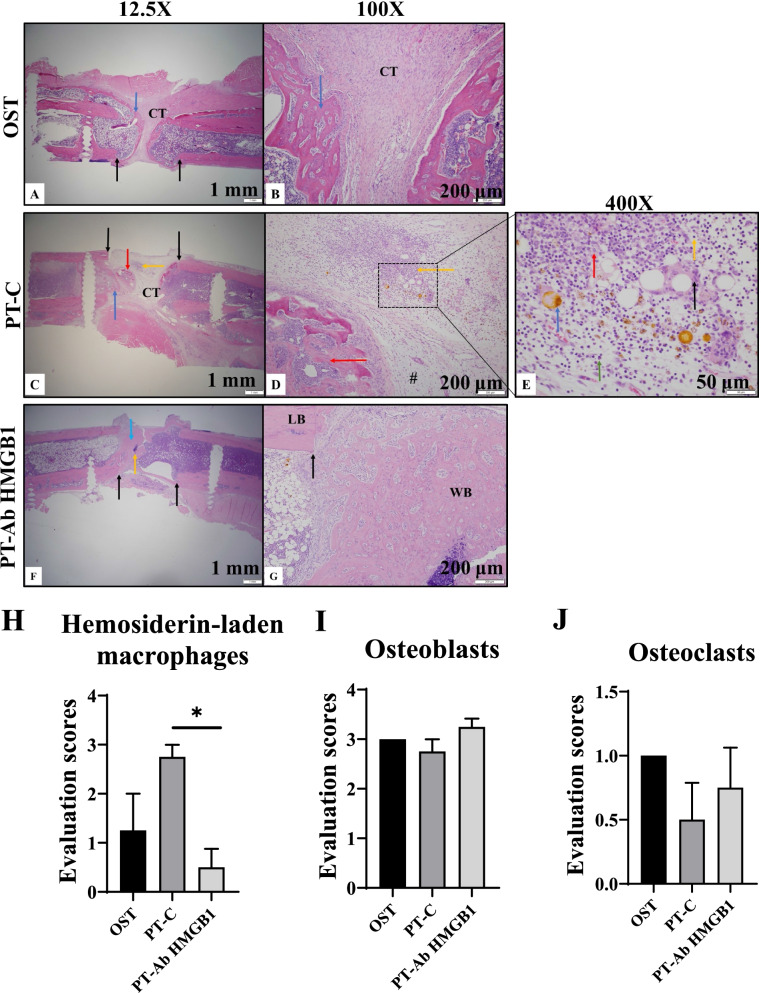
Osteotomy ‘defect’ site (black arrows) in a rat femur at 5 weeks post-trauma (wpt). **A** Hematoxylin and eosin (H&E) stained section of femur in the osteotomy group (OST). The callus of new bone growth (blue arrow) and fibrous connective tissue (CT) (12.5x). **B** Higher magnification of image A (100x). **C** Section of femur in polytrauma control (PT-C) group. The callus consists of new bone growth (blue arrow) and immature connective tissue (CT). Note the island of woven bone and bone marrow (red arrow) and focus of inflammation (yellow arrow) (12.5x). **D** Higher magnification of C (100x). **E** Higher magnification Figure C (400x). The focus of inflammation consists of multinucleated giant cells (black arrow), macrophages (red arrow), plasma cells (green arrow), lymphocytes (yellow arrow), and hemosiderin-laden macrophages (blue arrow) (chronic hemorrhage). **F** Section of femur in polytrauma + anti-HMGB1 antibody (PT-Ab HMGB1) group. The majority of the callus consists of new bone growth (blue arrow) with cartilage formation (yellow arrow). **G** Higher magnification of Figure J (100x). Compare the immature, woven bone (WB) in the callus to the mature, lamellar bone of the femur (LB). Graphical representation of the presence of **H** hemosiderin-laden macrophages; **I** osteoblasts; and **J** osteoclasts within the defect at 5 wpt in OST, PT-C, and PT-Ab HMGB1 rats (*n* = 4–8/group). The scoring key for hemosiderin-laden macrophages, osteoblast and osteoclasts is mentioned in the material and methods section

New bone growth was histologically evaluated using H&E stain at 5 wpt to score the bone/callus formation and evaluate cellular composition within the defect of OST, PT-C, and PT-Ab HMGB1 rats. The scoring key used to assess callus quality is mentioned in the materials and methods section. Histological evaluation validated enhanced bone regeneration in OST and PT-Ab HMGB1 compared to PT-C (Fig. 3A-G). The loose connective tissue rich in adipocytes in PT-C (Fig. 3D and E) was comparable with the more mature, densely cellular, fibrous connective tissue in OST (Fig. 3B). An increase in hemosiderin-laden macrophages were reported to be observed as focal accumulations at sites of old hematomas in diseased conditions signifying trauma-induced hemorrhage [25]. Hemorrhage can be acute (erythrocytes) or chronic (hemosiderin-laden macrophages). Acute hemorrhage occurs as soon as a vessel has ruptured. Hemosiderin is a break down product of heme and macrophages engulf the heme to remove it from the tissue. In severe trauma, the breakdown of erythrocytes takes a long time, during which hemosiderin-laden macrophages are present, and is characterized as chronic [26]. Chronic hemorrhage characterized by the presence of hemosiderin-laden macrophages was observed within the fracture defect of PT-C compared to OST and PT-AbHMGB1 (Fig. 3E and H). Although the osteoclasts and osteoblasts were slightly less in PT-C compared to OST and PT-AbHMGB1, there were no statistically significant differences at 5 wpt (Fig. 3I and J).

### Qualitative assessment of collagen content in the defect

**Fig. 4:**
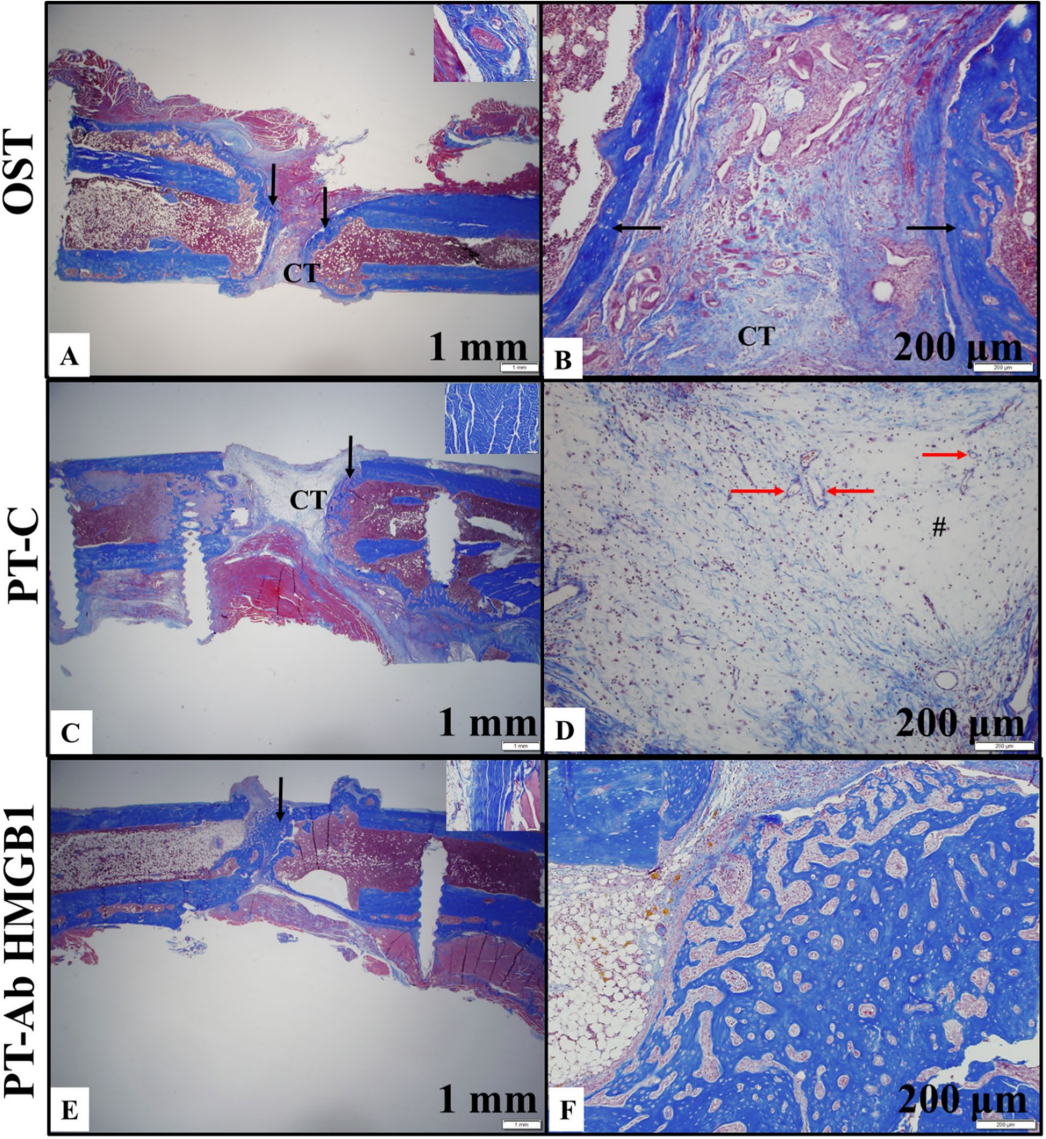
Decalcified sections of femurs 5 weeks post-trauma (wpt) were stained with Masson’s Trichrome (MT) stain. **A** Section of femur from the osteotomy (OST) group (*n* = 4). **B** Higher magnification of image A (100x). **C** Section of femur from the polytrauma (PT-C) group (*n* = 4). **D** Higher magnification of Figure C (100x). **E** Section of rat femur from the polytrauma + Anti-HMGB1 antibody (PT-Ab HMGB1) group (*n* = 8). **F** Higher magnification of Figure E (100x). Black arrows indicate new bone growth; red arrow indicates new blood vessels; CT – connective tissue

Originating from mesenchymal stem cells, the osteoblasts produce the organic components of the bone extracellular matrix, i.e., type I collagen fibers that, along with cells, make up the connective tissue. Masson’s Trichrome stain was used to evaluate organized collagen, which is seen by a deep saturation, to determine the connective tissue's quality, indicating mature connective tissue, which further suggests normal bone regeneration. In contrast, light saturation and sparse cells indicate immature connective tissue and poor bone regeneration. We noticed that at 5 wpt, in OST rats, the callus consists of new bone growth and fibrous connective tissue (Figs. 4A and B). In Fig. 4A, compare the immature fibrous connective tissue in the callus (400x) to the inset showing a mature connective tissue surrounding an artery. The connective tissue in the callus is moderately stained blue with partly organized collagen fibers compared to the mature connective tissue, stained deep blue, with densely packed organized collagen fibers (Fig. 4B). In the PT-C rats, the callus consists of lesser new bone growth and more immature fibrous connective tissue than the femur in the OST group (Figs. 4C and D). In Fig. 4C, compare the immature fibrous connective tissue in the callus (400x) to the inset showing a mature connective tissue of a tendon attached to the femur. The connective tissue in the callus is light blue, with more cells and sparse, disorganized collagen fibers compared to the mature connective tissue, stained deep blue, that has fewer cells, and densely packed organized collagen fibers (Fig. 4D). In PT-Ab HMGB1 rats the collagen fibers were distinctively denser, with mature connective tissue indicated by deep saturation compared to PT-C whose connective tissue, indicated by light saturation, had more cells and showed sparse disorganized collagen fibers. Majority of the callus consists of new bone growth (Fig. 4E and F). There was no qualitative difference between the intensity of staining between immature, woven bone and mature, lamellar bone and the mature connective tissue in a tendon attached to the femur as shown in the inset (Fig. 4E). Collectively, these observations confirm the quality of collagen in the extracellular matrix that determines the form and function of the bone.

### HMGB1 promotes γδTCR^+^ T cells depletion at the fracture site in PT

Since PT-Ab HMGB1 rats displayed increased bone formation than PT-C rats, we decided to profile the myeloid and lymphoid cells infiltrating the fracture site of OST, PT-C, PT-IgY, and PT-Ab HMGB1 rats and determine if HMGB1 had a role in modulating a dysregulated immune response at the injury site. At 1 wpt, cells from the fracture site were collected and stained for immunophenotyping. On average, we harvested ~ 3 million cells from the fracture site. Immunophenotyping analysis of immune cells revealed a slightly increased number of CD3^−^CD45^+^ leukocytes in PT-C rats and PT-IgY rats than OST and PT-Ab HMGB1 at 1 wpt, however, not statistically significant (Supplementary Figure S-2A). No differences in macrophages/Mɸ (i.e., CD3^−^CD45^+^CD68^+^CD86^+^ (M1) and CD3^−^CD45^+^CD68^+^CD163^+^ (M2)) occurred between all groups (Supplementary Figure S-2B and C).

**Fig. 5:**
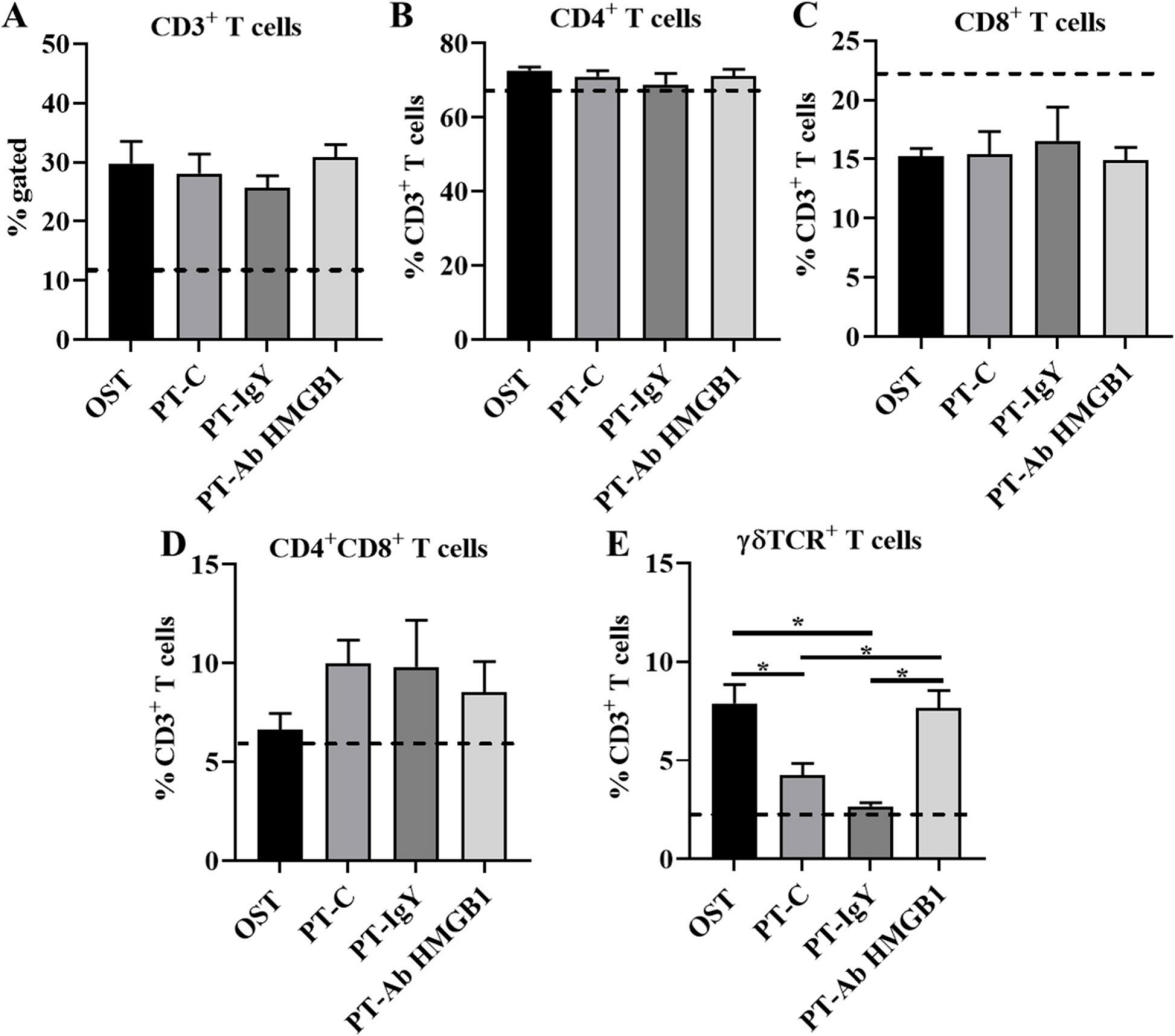
Immunophenotyping of immune cells in the fracture site at 1 week post-trauma (wpt) in osteotomy rats (OST), polytrauma rats (PT-C), PT rats with chicken IgY isotype (PT-IgY), and PT rats with anti-HMGB1 (PT-Ab HMGB1) rats. Frequencies of **A** CD3^+^ T cells in total; **B** CD3^+^CD4^+^ T cells; **C** CD3^+^CD8^+^ T cells; **D** CD3^+^γδ TCR^+^ T cells; **E** CD3^+^CD4^+^CD8^+^ T cells, all within the CD3^+^ T cells’ pool in OST, PT-C, PT-IgY and PT-Ab HMGB1 rats (*n* = 4–5/group). Bone marrow cells from naïve uninjured rats were used as baseline controls (*n* = 4) (dotted line). * *p* < 0.05 comparing OST, PT-IgY, PT-Ab HMGB1 and PT-C cell percentages. Data are graphically represented as mean ± SEM

Among the lymphoid cells, the αβT cells and the γδT cells were quantified in the fracture site at 1 wpt and the cell counts are listed in Table 2. There were no differences in CD4^+^ T cell, and CD8^+^ T cell percent counts across all groups (Fig. 5A-C). The percentage of CD4^+^CD8^+^ T cells were slightly increased in PT-C rats and PT-IgY rats than OST and PT-Ab HMGB1 at 1 wpt, however, there was nostatistical significance (Fig. 5D). The percent of γδ^+^TCR T cells decreased in PT-C (*p* = 0.01) and PT-IgY (*p* = 0.006) compared to OST. Interestingly, the percentage of γδ^+^TCR T cells was increased in the fracture site of PT-Ab HMGB1 compared to PT-C (*p* = 0.13) and PT-IgY (*p* = 0.04) (Fig. 5E).

**Table 2:** Relative counts of T cells in the fracture site at 1 week post-trauma (wpt) in osteotomy rats (OST), polytrauma rats (PT-C), PT rats with chicken IgY isotype (PT-IgY), and PT rats with anti-HMGB1 (PT-Ab HMGB1) rats. OST (*n* = 5), PT-C (*n *= 5), PT-IgY (*n* = 4) and PT-Ab HMGB1 (*n* = 8) rats, respectively. * *p* < 0.05 for OST vs PT-IgY and PT-C and ** *p* < 0.05 for PT-Ab HMGB1 vs PT-IgY and PT-C

T cells	Cell counts (Mean ± SD)			
	**OST**	**PT-C**	**PT-IgY**	**PT-Ab HMGB1**
CD3^+^ T cells	2233 ± 855	2052 ± 877	2072 ± 339	2038 ± 536
CD3^+^ CD4^+^ T cells	1632 ± 650	1446 ± 580	1393 ± 338	1458 ± 418
CD3^+^ CD8^+^ T cells	344 ± 154	331 ± 224	334 ± 117	301 ± 232
CD3^+^ CD4^+^ CD8^+^ T cells	156 ± 82	208 ± 104	370 ± 77	173 ± 92
CD3^+^ γδTCR^+^ T cells	237 ± 104*	105 ± 36	96 ± 33	234 ± 57* *

## Discussion

This study aimed to establish the role of secreted HMGB1 in mediating immune dysregulation in PT associated with delayed fracture healing. The main findings of this study are that the PT-Ab HMGB1 rats demonstrated increased fracture healing with enhanced bone formation within the fracture defect compared to PT-C rats at 5 wpt. Additionally, severe depletion of γδTCR^+^ T cells counts was observed in PT-C and PT-IgY rats compared to OST. Interestingly, the γδTCR^+^ T cells counts remained significantly elevated at 1 wpt in PT-Ab HMGB1 and OST rats.

In the context of oral bone regeneration, HMGB1 has a beneficial role due to it’s ability to initiate an acute inflammatory response to activate regenerative pathways [9, 21]. While some studies have reported the osteogenic role of HMGB1 in mediating bone regeneration of fractures in single injury models [16, 27, 28], some other studies have also suggested its detrimental role in impairing tissue regeneration potentiating remote organ injury [10, 29]. Yet, some others have recently presented the exogenous supplementation of HMGB1 at the fracture site to promote bone regeneration, demonstrating that HMGB1 primes MSCs for osteogenic differentiation [30, 31]. However, the findings from a series of experiments conducted by Lee et al. clarified the confusion about the role of HMGB1 in fracture healing, demonstrating that HMGB1 only in its fully reduced form (fr-HMGB1) has regenerative properties as opposed to its disulfide form (ds-HMGB1) [19]. They further suggest that the post-translational modifications of HMGB1 determines its role in inflammation and immunity. Further, Lee et al. also demonstrated that complete blocking of HMGB1 with glycyrrhizin resulted in delayed fracture healing [31]. We posit that while secreted HMGB1 is needed for regulating bone regeneration, its regenerative function is primarily concentration-dependent. To our knowledge, this is the first study to assess bone regeneration following neutralization of early-secreted HMGB1 immediately following injuries in a polytrauma rat model.

It is clear structural reconstitution of bone healing after fractures relies on both innate and adaptive immune responses during the entire inflammatory phase. If the immune cell kinetics are disturbed, the essential immune cells required for callus formation become unavailable, thereby delaying or impairing bone healing. As for normal fracture healing, the inflammatory phase begins at the time of injury and lasts up to 5 days, followed by the soft callus formation phase. However, in the event of delayed fracture healing, the inflammatory phase extends to > 5 dpt reviewed in [15, 32]. Khassawna et al. demonstrated that T cells, rather than B cells, contributed to improved bone extracellular matrix deposition, collagen organization, and fracture healing at 1 wpt [6]. Fractured bones from mice lacking mature T cells were densely mineralized and stiffer but significantly compromised bone quality compared to the fractured bones from mice with normal T cell counts. This phenotype correlated well with collagen deposition and osteoblast distribution within the fracture site [6, 33]. Since the impact of T cells and their subtypes on fracture repair is gaining much attention, and because HMGB1 functions as a central cytokine for all lymphoid cells, we proceeded to capture a snapshot of the αβ T cells and γδ T cells present in the fracture space at 1 wpt. While the CD4^+^ T cells and CD8^+^ T cell counts within the CD3^+^ T cell pool in the fracture space were not comparable between all groups, there was an increasing trend in the CD4^+^CD8^+^ (double-positive) T cells in PT-C and PT-IgY rats, but this increase was not statistically significant. The double-positive T cells express αβTCRs and they share the features of both CD4^+^ as well as CD8^+^ T cells. They have been previously found enriched in tissues at the site of inflammation in various pathological settings, including cancers and rheumatoid arthritis, [17, 34, 35]. However, CD4^+^CD8^+^ double-positive T cells' role is mostly understudied in trauma. Their function remains controversial, with conflicting reports describing cytotoxic and suppressive roles for these cells, and is an ongoing debate. This study is the first report describing CD4^+^CD8^+^ double-positive T cells in the long bone fractures. Further research is needed to assess the possible involvement of double-positive T cells in the fracture site's immune surveillance and bone regeneration in PT.

The γδTCR^+^ T cells are prevalent at the injury sites and are known for their immune surveillance role with a final goal to enhance healing [20]. Colburn et al. have reported a particular pattern in the γδTCR^+^ T cell dynamics at the fracture site that delays the healing cascade, a phenotype possibly occurring due to the heavy oxidative burst that prevails at the injury site [19]. Other investigations emphasized the importance of the γδTCR^+^ T cells in bone regeneration, demonstrating a strong potential of these cells to serve as precursors of possible adverse immune effects of immunosuppressive therapies [36, 37]. Further, in vitro investigations demonstrated that upon activation, the γδTCR^+^ T cells tend to inhibit osteoclastogenesis and promote osteoblastogenesis via the secretion of specific growth factors and cytokines [38]. We infer from our findings that despite the inconsequential role of CD4^+^ and CD8^+^ T cells in polytraumatic fractures, the innate-lymphocytes, particularly the γδTCR^+^ T cell, may have a potential role during fracture healing. These findings are as per the previous reports demonstrating that it is not the αβT cells, but rather, the γδT cells are essential players of ossification and bone regeneration following fractures [36]. However, fundamental information about the biological link between the γδTCR^+^ T cells and HMGB1 in the context of polytraumatic fracture healing needs further elucidation.

The limitation of this study is that it represents a snapshot of the immune cell profile at 1 wpt, which may change dynamically over time. This time-point was chosen because of the prolonged inflammatory phase in delayed fracture healing, and immune cell infiltration that was detected at 1 week in previous fracture repair studies [6]. Evaluating multiple time points earlier and later than 1 week would explain whether the magnitude and phenotypic distribution of γδTCR^+^ T cell dynamics evolves temporally at the fracture site after multiple concurrent injuries. Additionally, since there was no difference in cellular distributions in the PT-IgY group compared to PT-C, bone healing was evaluated using the PT-C group as the control group. Finally, toward the ultimate goal of therapeutic immunomodulation, follow-up studies should employ gain- and loss-of-function techniques to elucidate the functions of γδTCR^+^ T cells with and without neutralizing extracellular HMGB1 in PT.

## Conclusion

In concordance with the previously published reports on T cells in fracture healing, we showed that PT did not significantly influence αβ T cell frequencies; instead, it decreased γδTCR^+^ T cell frequencies within the fracture space. Following neutralization of secreted HMGB1 post-polytraumatic injuries in rats, we established a role for HMGB1 in bone regeneration. We conclude that by regulating extracellular HMGB1 levels, the bone formation process was guided to allow for proper ossification leading to improved bone quality and resolution accompanied by an increase in γδTCR^+^ T cells at the fracture site. Moving forward, by selectively targeting danger molecules such as HMGB1 either locally or systemically with potential therapeutic biologics, new interventions may determine efficient regulation of deranged immune responses to promote fracture repair in PT patients.

## Supplementary Information

**Additional file 1. **Micro-computed tomography (μCT) analysis results of bone regeneration in polytrauma (PT) rats following treatment with three doses (1dose/day 0, 1 and 2) of anti-HMGB1 antibody (PT-HMGB1 3x). 

**Additional file 2.** HMGB1 increases infiltration of myeloid cells at the fracture site in polytrauma (PT).

## Data Availability

The datasets generated during and/or analyzed during the current study are available from the corresponding authors on reasonable request.
