# Supplementary material for: Neutralization of HMGB1 improves fracture healing and γδ T lymphocyte counts at the fracture site in a polytrauma rat model
Source: J Exp Orthop. 2022 Feb 28;9:21. doi: 10.1186/s40634-022-00453-3 (PMC8885932; doi:10.1186/s40634-022-00453-3)
Supplement: Supplementary file 1 — Additional file 1. Micro-computed tomography (μCT) analysis results of bone regeneration in polytrauma (PT) rats following treatment with three doses (1dose/day 0, 1 and 2) of anti-HMGB1 antibody (PT-HMGB1 3x). [file 40634_2022_453_MOESM1_ESM.pdf]

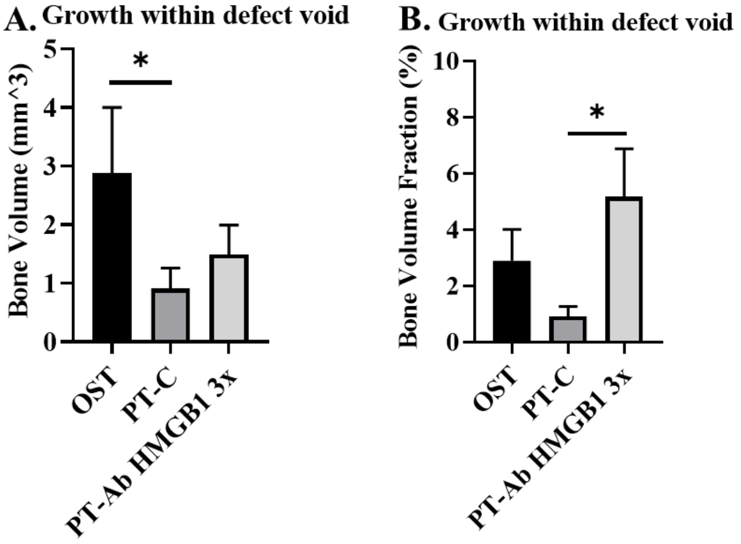

Supplementary Figure S1: Micro-computed tomography (μCT) analysis results of bone regeneration in polytrauma (PT) rats following treatment with three doses (1dose/day 0, 3 and 7) of anti-HMGB1 antibody (PT-HMGB1 3x). (A) Bone Volume (mm^3) results from the Defect volume of interest (VOI) and (B) Bone Volume Fraction (%) results from the Defect VOI. Osteotomy (OST), b) Polytrauma (PT-C), c) Polytrauma + AbHMGB1 3x doses (PT-HMGB-1 3x) (n=6-10/group). * $p<0.05$ versus corresponding non-treated PT group (PT-C). Data are graphically represented as mean± SEM.
