# Supplementary material for: Neutralization of HMGB1 improves fracture healing and γδ T lymphocyte counts at the fracture site in a polytrauma rat model
Source: J Exp Orthop. 2022 Feb 28;9:21. doi: 10.1186/s40634-022-00453-3 (PMC8885932; doi:10.1186/s40634-022-00453-3)
Supplement: Supplementary file 2 — Additional file 2. HMGB1 increases infiltration of myeloid cells at the fracture site in polytrauma (PT). [file 40634_2022_453_MOESM2_ESM.pdf]

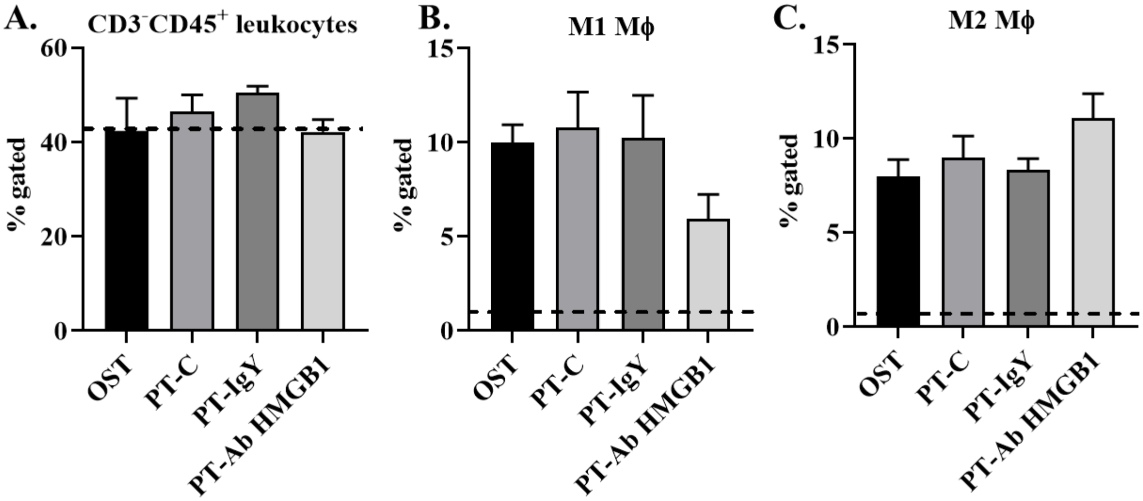

Supplementary Figure S2: HMGB-1 increases infiltration of myeloid cells at the fracture site in PT. Immunophenotyping of myeloid cells in the wound space (WS) at 1 week post-trauma (wpt) in osteotomy rats (OST), polytrauma rats (PT-C), PT rats with chicken IgY isotype (PT-IgY), and PT rats with anti-HMGB1 (PT-Ab HMGB1) rats. WS includes the hematoma/soft callus from the fracture defect site and a rinse of the area around the defect. Frequencies of (A) $CD3^-CD45^+$ leukocytes in total; (B) $CD3^-CD45^+CD68^+CD86^+$ (M1 Macrophages (Mϕ)); (C) $CD3^-CD45^+CD68^+CD163^+$ (M2 Mϕ) in OST, PT-C, PT-IgY and PT-Ab HMGB1 rats, respectively (n=4-5/group). Bone marrow cells from naïve uninjured rats were used as baseline controls (n=5) (dotted line). * $p<0.05$ comparing OST, PT-IgY, and PT-Ab HMGB1 count to PT-C counts. Data are graphically represented as mean ± SEM.
